# A bibliometric analysis of myocardial bridge combined with myocardial infarction

**DOI:** 10.1097/MD.0000000000038420

**Published:** 2024-06-07

**Authors:** Haiyuan Zhang, Yuejuan Cao

**Affiliations:** a School of Graduate, Tianjin University of Traditional Chinese Medicine, Tianjin, China; b Department of Cardiology, Tianjin Union Medical Center, Tianjin, China.

**Keywords:** bibliometrics, myocardial bridges, myocardial infarction, visual analysis, Web of Science

## Abstract

**Background:**

The aim of this study is to analyze the process and frontiers of research in myocardial bridges (MB) to identify future research directions in the last 3 decades.

**Methods:**

Relevant literature on MB combined with myocardial infarction (MI) was searched from 1991 to 2023 in the Web of Science database, and was analyzed by bibliometric analysis using VOSviewer, CiteSpace, and the R package “bibliometrix.”

**Results:**

A total of 1233 English articles were included in this study. The number of published articles showed an increasing trend yearly. From 2017 to 2022, the annual publication volume rose rapidly, and in 2021 the publication volume even reached 95 articles, which was the highest in all years. These publications were from 68 countries and 1854 institutions, with the leading country being the U.S. and the leading institution being Columbia University. Myoho Clinical International has a close collaborative relationship with Columbia University, while in recent years, the Harvard Medical School has explored the study of MB combined with MI. *Annals of Thoracic Surgery* was the journal with the highest number of publications, and Takayama Hiroo and Naka Yoshifumi were the authors with the highest number of publications. The most common keywords were MI, cardiogenic shock, and MB.

**Conclusions:**

Our findings can help researchers explore the current status of MB combined with MI research and choose new survey routes for upcoming studies. Prevalence and prognosis, mechanism of MB combined with MI and molecular mechanism may become the focus of future research. In addition, more research and cooperation are needed worldwide.

## 1. Introduction

Myocardial bridges (MB) are congenital anomalous changes in the vasculature. Normally, the coronary arteries and their branches travel on the surface of the heart and are covered by the epicardium. However, in some cases, a portion of the coronary arteries is covered by superficial myocardial fibers, and the covered vessel is called the mural or tunnel artery, and the myocardium covering the mural arteries is called MB.^[1]^ This particular variant was discovered by Reyman in 1737 at autopsy and was first described by Portmann and Iwig in 1960 by angiography.^[2]^ Studies have confirmed that 67% to 98% of MB occur in the mid-left anterior descending branch,^[3,4]^ but they can also occur in any epicardial coronary artery such as the posterior descending branch of the right coronary artery, the posterior interventricular branch or the spinous branch.^[5]^ Based on their anatomical morphology, MB can be categorized into superficial MB located in the interventricular groove and deep MB located close to the right ventricular septum.^[6]^ Currently, MB is considered a benign anatomical variant, but some significant concomitant symptoms have been reported in the literature, such as stable angina pectoris, myocardial infarction (MI), malignant arrhythmias, and even sudden cardiac death.^[7]^ In terms of treatment, pharmacologic therapy is currently preferred for most cases, while surgical treatments such as percutaneous coronary intervention, supra-arterial myotomy and coronary artery bypass grafting (CABG) are considered in patients with refractory angina pectoris.^[8]^

Bibliometrics is currently a popular method to analyze the literature, which can deeply analyze the research hotspots and trends in a certain field, and conduct a comprehensive study on the information of authors, keywords, journals, countries, references, etc,^[9]^ and therefore it is widely used in the fields of oncology,^[10]^ orthopedics,^[11]^ cardiovascular medicine^[12]^ and so on. However, according to the survey, there is no bibliometric analysis of MB combined with MI, so we cannot accurately grasp the current status of its research. In this study, we used bibliometrics to summarize the current research status of MB combined with MI, analyze the research hotspots in this field, and find out its trend for future development. Our goal was to use this approach to assess the state of the art and new directions in MB-related study and to offer an in-depth analysis of the field state of development for researchers to refer to for future work.

## 2. Materials and methods

The Web of Science Core Collection (WoSCC) database was searched for literature on MB combined with MI, setting the subject terms as “myocardial bridge” and “myocardial bridge” and “myocardial infarction,” time span: January 1, 1991 to June 30, 2023. A total of 1463 articles were included. After removing conference proceedings, early access, books, patents, and documents with little relevance by using the labeling type of the database and setting the language of the documents as “English,” a total of 1233 documents were finally included in the database, and then exported in plain text format and named as “download_txt.” The study protocol was approved by the review board of Tianjin Union Medical Center.

VOSviewer (version 1.6.18) is the current bibliometric analysis software that can extract important information from the literature to be searched. It is commonly used to map collaborative, co-cited networks.^[13]^ In this study, the software was used to accomplish the following 5 analyses: country and institutional analysis, journals and co-cited journals analysis, authors and co-cited authors analysis, co-cited references analysis, and keyword co-occurrence analysis. In the network drawn by VOSviewer, each node represented 1 element, such as a country, a journal, an author, or an institution, while the size and color of the nodes represented the number and classification of these items. In addition, interconnections between the points reflected the relationship of co-citation, and the thickness of the cooperation appeared to increase with the number of interconnections, representing the strength of the link.^[14]^

CiteSpace (version 6.2.R4) is another bibliometric analysis software developed by Prof Chaomei Chen.^[15]^ In this study, this software was applied to the dual-map overlay analysis and burst citation literature analysis of journals. The R package “bibliometrix” (version 3.2.1) (https://www.bibliometrix.org) is also a bibliometric analysis software,^[16]^ which was used in the present study group to construct a global distribution network of publications on MB. In addition, we applied Microsoft Office Excel 2019 to quantitatively analyze the publication of the literatures.

## 3. Results

### 3.1. Quantitative analysis of publication

**Figure 1.**
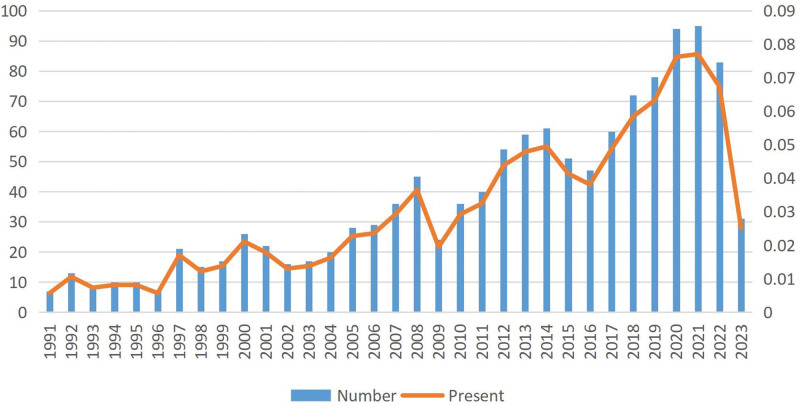
Trends in the growth of publications worldwide from 1991 to 2022.

The analysis of the annual number of publications can grasp the research trends and hot spots in this field. As shown in Figure 1, the annual publication volume on MB combined with MI was at a low level from 1991 to 2004, with an average of 15 publications per year. In the following 12 years, the amount of literature published on related studies increased, with an average of 42 articles per year. From 2017 to 2022, the annual publication volume rose rapidly, and in 2021 the publication volume even reached 95 articles, which was the highest in all years. These results showed that more and more researchers have focused on MB combined with MI, with a view to understanding its physiology, pathology, and treatment in more details.

### 3.2. Country and institutional analysis

**Figure 2.**
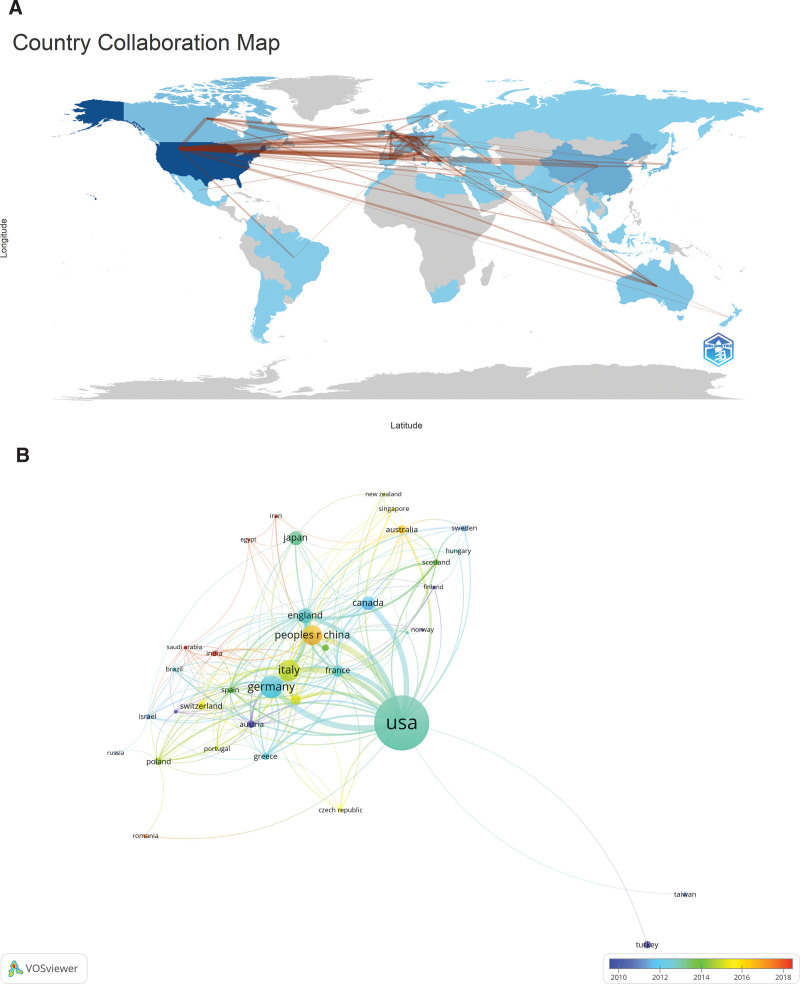
The geographical distribution (A) and visualization of countries (B) on research of myocardial bridges combined with myocardial infarction.

Through the analysis, it can be concluded that these articles come from 68 countries and 1854 organizations, and the specific results are shown in Table 1. Among them, the top 10 active countries are distributed in Asia, North America and Europe, and the largest number of articles is from the United States (n = 464, 37.6%), followed by Germany (n = 129, 10.5%), Italy (n = 116, 9.4%) and China (n = 107, 8.7%), and the number of articles from the U.S. and Germany accounts for about half of the total number (48.1%). Subsequently, the number of publications was adjusted to be greater than or equal to 5, and a collaborative network was constructed for the partnership among 37 countries and the geographical distribution was compared (Fig. 2). It was obvious to see that there was a close cooperation among countries in the world, such as between the United Kingdom and Italy, and the United States has a very tight partnership with Germany and China.

**Table 1:** Top 10 productive countries and institutions.

Rank	Country	Counts	Institution	频次
1	The United States	464(37.6%)	Columbia University(USA)	27(2.1%)
2	Germany	129(10.5%)	Myoho Clinical International(USA)	23(1.9%)
3	Italy	116(9.4%)	Duke University(USA)	19(1.5%)
4	China	107(8.7%)	Harvard University(USA)	17(1.4%)
5	England	71(5.8%)	Stanford University(USA)	16(1.3%)
6	Japan	64(5.2%)	University of Milan(Italy)	15(1.2%)
7	Canada	62(5.0%)	Ohio State University(USA)	15(1.2%)
8	France	48(3.9%)	Cleveland Clinic(USA)	15(1.2%)
9	Netherlands	38(3.1%)	University Hospital Zurich(Germany)	13(1.1%)
10	Australia	30(2.4%)	University of California Los Angeles(USA)	13(1.1%)

**Figure 3.**
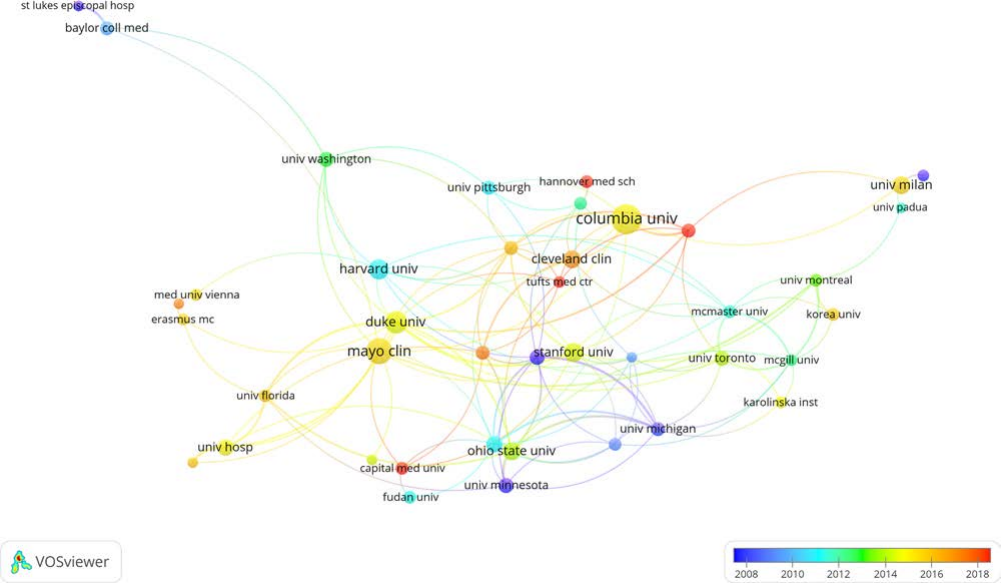
The visualization of institutions on research of myocardial bridges combined with myocardial infarction.

The vast majority of the top 10 research institutions were in the United States, such as Columbia University (n = 27, 2.1%), Myoho Clinical International (n = 23, 1.9%), Duke University (n = 19, 1.5%), and Harvard University (n = 17, 1.4%) that has the highest number of publications. As shown in Figure 3, the minimum number of publications was adjusted to 8, so as to establish a collaborative network among the 41 institutions. Myoho Clinical International has a close collaborative relationship with Columbia University, while in recent years, the Harvard Medical School has explored the study of MB combined with MI.

### 3.3. Journals and co-cited journals analysis

**Figure 4.**
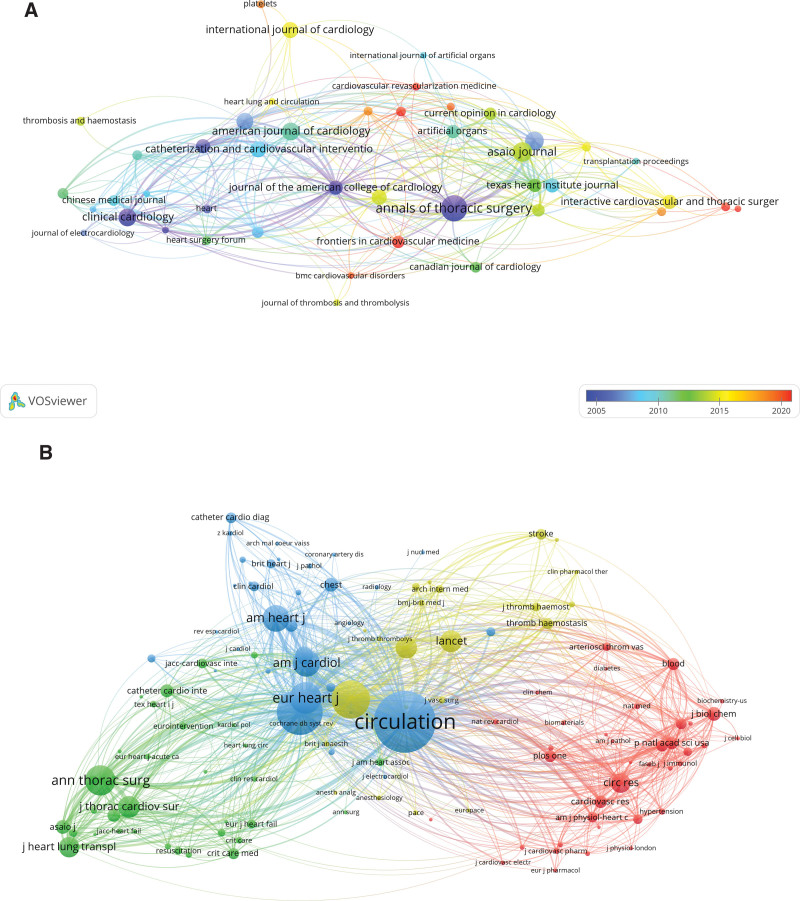
The visualization of journals (A) and co-cited journals (B) on research of myocardial bridges combined with myocardial infarction.

According to the statistics, articles related to research on MB combined with MI were published in 511 journals. As shown in Table 2, *Annals of Thoracic Surgery* (28 articles) was in first place among the top 10 referenced journals, followed by *Asaio Journal* (n = 20, 1.6%), *Journal of Heart and Lung Transplantation* (n = 19, 1.5%), *Clinical Cardiology* (n = 18, 1.5%). Among the top 10 journals, the highest impact factor was *Circulation* (IF = 37.8), followed by *Journal of the American College of Cardiology* (IF = 24.0). Subsequently, the minimum number of publications was changed to 6, and 44 journals were screened and visualized to create a journal network, where *Annals of Thoracic Surgery* and *Cardiovascular Revascularization Medicine, Circulation* all have a very close citation relationship (Fig. 4A).

**Table 2:** Top 10 journals and co-cited journals for research of myocardial bridges combined with myocardial infarction.

Rank	Journal	Counts	IF	Q	Co-cited Journal	Counts	IF	Q
1	Annals of Thoracic Surgery	28(2.3%)	4.6	Q2	Circulation	3821	37.8	Q1
2	Asaio Journal	20(1.6%)	4.2	Q2	Journal of the American Collage of Cardiology	2427	24.0	Q1
3	Journal of Heart and Lung Transplantation	19(1.5%)	8.9	Q1	New England Journal of Medicine	1841	158.5	Q1
4	Clinical Cardiology	18(1.5%)	2.7	Q3	European Heart Journal	1398	39.3	Q1
5	American Journal of Cardiology	18(1.5%)	2.8	Q3	Annals of Thoracic Surgery	1316	4.6	Q2
6	Circulation	17(1.4%)	37.8	Q1	American Journal of Cardiology	1117	2.8	Q3
7	Catheterization and Cardiovascular Interventions	16(1.3%)	2.3	Q3	American Heart Journal	1078	4.8	Q2
8	International Journal of Cardiology	16(1.3%)	3.5	Q2	Lancet	791	168.9	Q1
9	Journal of Cardiac Surgery	15(1.2%)	1.6	Q4	Journal of Thoracic and Cardiovascular Surgery	788	6.0	Q1
10	Journal of the American Collage of Cardiology	14(1.1%)	24.0	Q1	JAMA-Journal of the American Medical Association	781	120.7	Q1

Among the top 10 cited journals, *Circulation* (n = 3821) was in first place among the top 10 referenced journals, followed by *Journal of the American College of Cardiology* (n = 2427), *Annals of Thoracic Surgery* (n = 1935), *New England Journal of Medicine* (n = 1841), and *New England Journal of Medicine* (n = 1841). *Lancet* had the highest impact factor (IF = 168.9), followed by *New England Journal of Medicine* (IF = 158.5), *JAMA-Journal of the American Medical Association* (IF = 120.7). The minimum number of citations was set at 50 to create a co-citation network, and the results were shown in Figure 4B, where *Circulation* was co-cited with *European Heart Journal, American Journal of Cardiology*, and so on.

**Figure 5.**
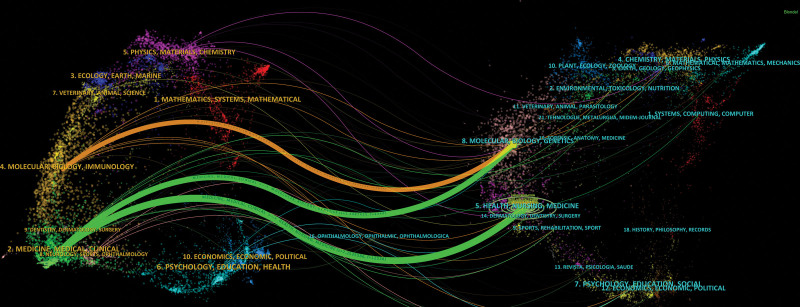
The dual-map overlay of journals on research of myocardial bridges combined with myocardial infarction.

In Figure 5, we performed a dual-map overlay of journals on MB combined with MI with CiteSpace to show the citation relationships of journals. The citing journals cluster was displayed on the left and the cited journals cluster was on the right. In addition, the orange path indicated that literature published in molecular/biology/genetics journals is mainly cited by literature in molecular/biology/immunology journals, while the green path indicated that literature published in molecular/biology/genetics and health/nursing/pharmacy journals are mainly cited by literature in pharmacy/clinical medicine journals.

### 3.4. Authors and co-cited authors analysis

**Figure 6.**
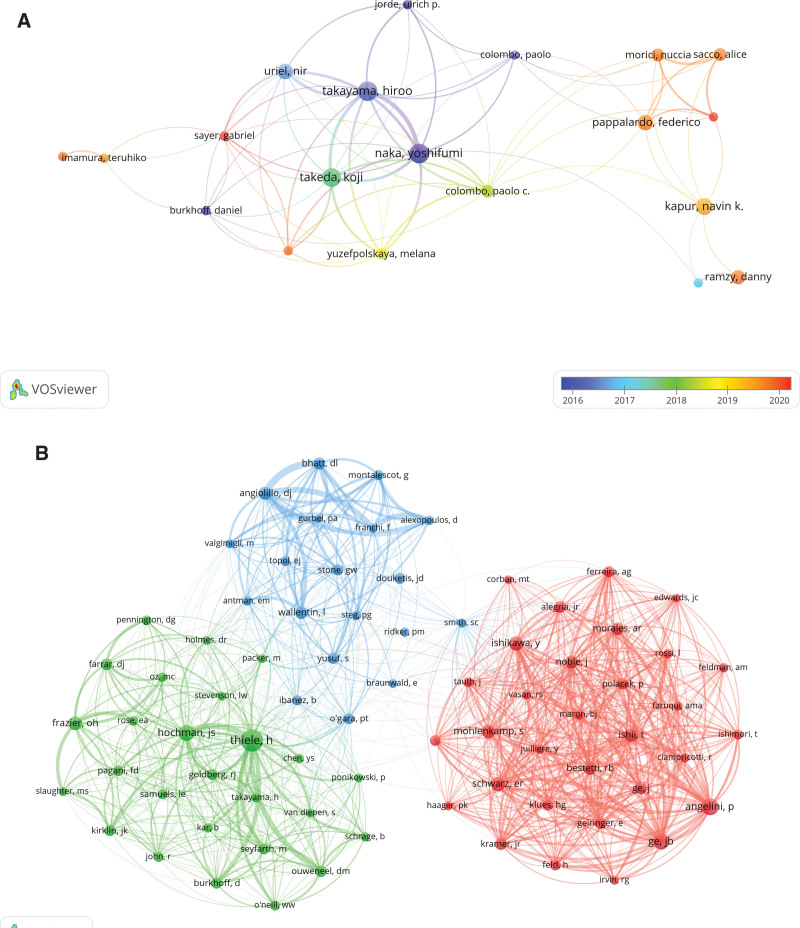
The visualization of authors (A) and co-cited authors (B) on research of myocardial bridges combined with myocardial infarction.

After statistics, there were 7055 authors involved in the study of MB combined with MI, among which 3 of the top 10 authors have published more than 10 pieces of related literature, as shown in Table 3. The minimum value of the number of published papers was set to 4, so as to build up the corresponding collaborative network, as shown in Figure 6A, the thicker the connecting line and the higher the density, which indicated that the more collaborations and the closer the connection, such as In recent years, Oliva Fabrizio has collaborated very closely with Morid Nuccia, Sacco Alice, Kapur Navin K., and others.

**Table 3:** Top 10 authors and co-cited authors for research of myocardial bridges combined with myocardial infarction.

Rank	Authors	Count	Co-cited authors	Citations
1	Takayama Hiroo	11	Thiele Holger	232
2	Naka Yoshifumi	11	Angelini Paolo	134
3	Takeda Koji	10	Junbo Ge	123
4	Kapur Navin K.	9	Hochman Judith S.	119
5	Uriel Nir	8	Schwarz Erika Reategui	96
6	Pappalardo Federico	8	Ishikawa Yojiro	96
7	Kar Biswajit	7	Moehlenkamp Stefan	92
8	Hagl Chridtian	7	Frazier O.H.	88
9	Ramzy Danny	7	Ishii Taku	84
10	Angiolillo Dominick J.	7	Angiolillo Dominick J.	80

There are a total of 26,901 co-cited authors, of which 4 have been cited more than 100 times, as shown in Table 3, and the author with the highest number of co-citations is Thiele Holger (n = 232), followed by Angelini Paolo (n = 134) and Junbo Ge (n = 123). Setting the number of co-citations to be greater than or equal to 35 to create a co-citation network, as shown in Figure 6B. The results showed that there was an equally active collaboration among co-cited authors, such as the close collaboration between Thiele Holger and Hochman Judith S.

### 3.5. Hotspots and frontier

**Figure 7.**
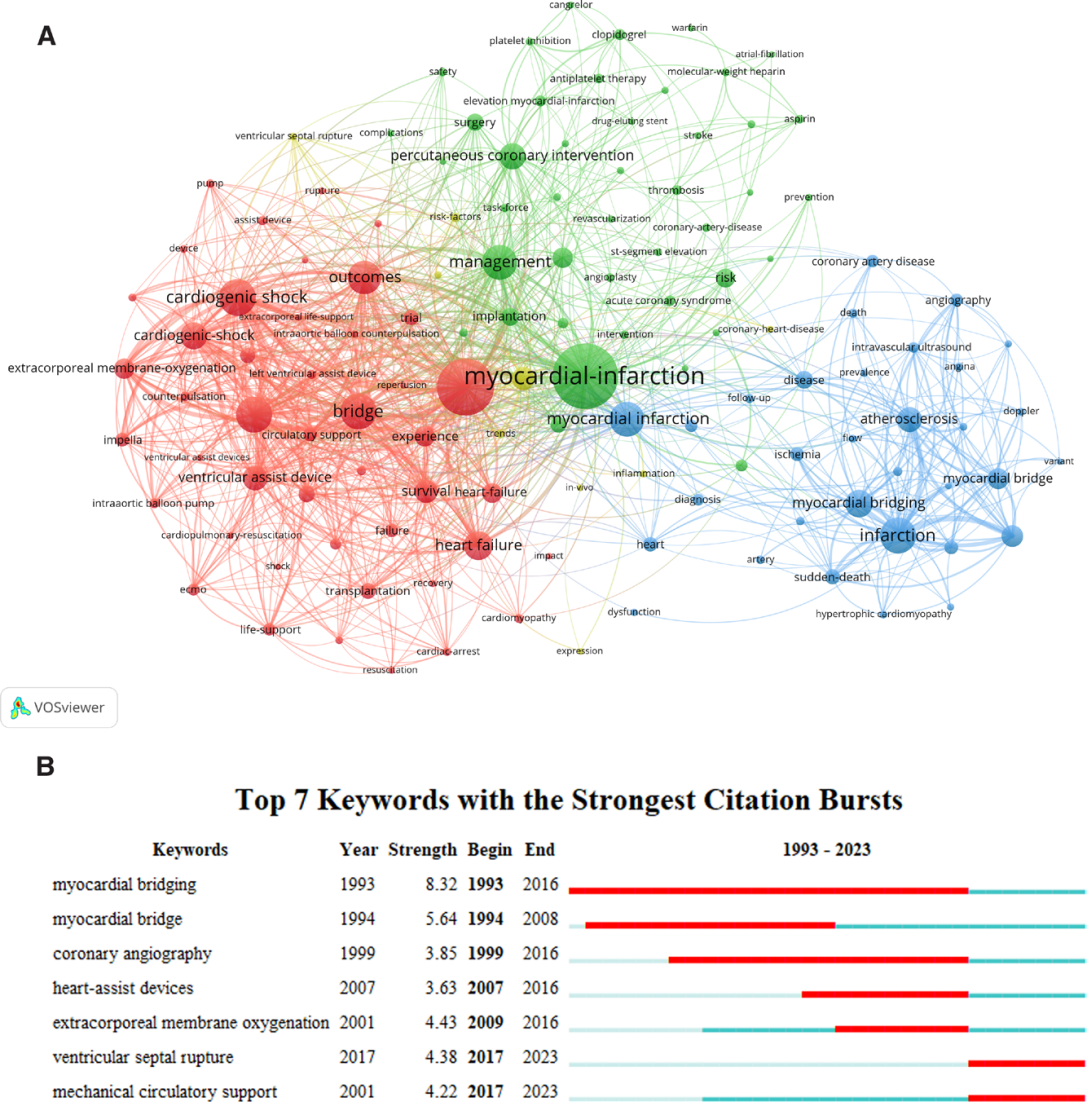
Top 20 keyword cluster analysis (A) and top 15 keywords with the strongest citation bursts (B).

By analyzing the keywords, it is possible to know the hot topics of the current researched field more accurately. In Table 4, the top 20 high-frequency keywords about MB were listed, and it can be seen that “myocardial infarction” and “cardiogenic shock” appeared more frequently, which is the main research direction of MB combined with MI at present. The keywords with a frequency more than 15 were screened for cluster analysis, and different colors represented different research directions, and the results were shown in Figure 7A, which yielded a total of 3 clusters, with the keywords in the green cluster being MI, percutaneous coronary intervention, and antiplatelet therapy, etc; the keywords in the red cluster being cardiogenic shock, extracorporeal life support, heart failure, etc; the keywords in the blue cluster being MB, left anterior descending branch, sudden death, etc.

**Table 4:** Top 20 keywords on research of myocardial bridges combined with myocardial infarction.

Rank	Keywords	Count	Rank	Keywords	Count
1	Myocardial infarction	267	11	Myocardial bridging	90
2	Acute myocardial infarction	225	12	Cardiogenic shock	88
3	Cardiogenic shock	130	13	Percutaneous coronary intervention	86
4	Bridge	127	14	Ventricular assist device	80
5	Mechanical circulatory support	126	15	Atherosclerosis	79
6	Infarction	126	16	Mortality	72
7	Management	122	17	Survival	72
8	Myocardial infarction	120	18	Descending coronary artery	67
9	Outcomes	114	19	Myocardial bridge	65
10	Heart failure	95	20	Experience	63

The keywords and emergent words detection were mainly used to analyze the keywords that were cited with a sudden increase in a certain time, so as to predict the cutting-edge research direction, and reflect the emerging academic trends and hotspots in a certain field. Figure 7B showed that the results of keyword emergence analysis of MB combined with MI research, and the red part on the blue timeline indicated the start year, end year and duration of emergence, which showed that “myocardial bridge” had the highest emergence intensity (8.32), followed by “extracorporeal membrane pulmonary oxygenation” (8.32), and “extracorporeal membrane pulmonary oxygenation” (8.32). membrane pulmonary oxygenation” (4.43), “septal perforation” (4.38), and “mechanically assisted circulation” (4.22).

## 4. Discussion

### 4.1. General information

In this study, we used VOSviewer, CiteSpace, and the R package “bibliometrix” software to visualize and analyze 1233 papers on MB combined with MI in the Web of Science database from 1991 to 2023. During the period of 1991~2004, the number of publications on MB combined MI research was at a low level, and then in the following 12 years, the research in this field gradually started, and the publication of related literature increased, and then the number of publications increased rapidly. In addition, MB combined MI became a popular research direction, which attracted more and more researchers’ attention. The United States and Germany are the major countries in this field, and most of the top 10 research organizations are from the United States. Among them, the United Kingdom and Italy are actively cooperating with each other. United States, China and Germany are also very close partnership, and in recent years, Harvard Medical School is cooperating with Stanford University and Duke University is particularly active. However, the intensity of cooperation between some countries and research institutions is not ideal, such as the lack of more cooperation between the United Kingdom and Portugal, which is detrimental to the development of the field in the long run. Therefore, we call for more countries and research institutions to work together to promote the further development of MB combined with MI research in a more in-depth manner.

Literature studies on MB combined with MI were published in the journal *Annals of Thoracic Surgery* (IF = 4.6, Q2) with the highest number, proving that this journal is the most popular journal by far. And the journal with the highest impact factor was *Circulation* (IF = 37.8, Q1), followed by *Journal of the American College of Cardiology* (IF = 24, Q1). The majority of the co-cited journals were high-impact Q1 journals that have published literature on MB combined with MI research that has contributed indelibly to the development of the field.

In terms of authors, Takayama Hiroo, Naka Yoshifumi, and Takeda Koji published the largest number of papers. Among them, Prof Takayama Hiroo, together with Prof Naka Yoshifumi and Prof Takeda Koji, published the most papers, and the main research content of them was on the role of cardiac assist devices of MB and MI and cardiogenic shock as well as cardiogenic shock.^[17–19]^

### 4.2. The hotspots and frontiers

Keywords can help us quickly uncover the distribution and evolution of research hotspots in a field. By summarizing and analyzing the above keyword clustering analysis and emergence analysis (Fig. 7), we concluded that the current research focus around the world was mainly in the following areas:

### 4.3. Pathology and pathophysiology

MB has long been recognized as a benign variant with a favorable long-term prognosis for patients, but many studies have demonstrated that they may still lead to a variety of adverse cardiac events, including MI, life-threatening arrhythmias, and sudden cardiac death,^[3,20,21]^ which are associated with the degree of MB compression, with the more MB are compressed, the higher the risk of adverse cardiovascular events the greater the risk of adverse cardiovascular events.^[22]^ Sung-Soo Kim et al^[23]^ observed 684 patients with MB suffering from persistent chest pain without severe stenosis by coronary artery examination at a 37-month follow-up. As a result, 79 developed refractory chest pain (11.5%), 8 developed MI (1.2%), 1 developed life-threatening arrhythmia (0.1%), and 4 died (0.6%). The causes of MB leading to MI have not been investigated, and the studies preferred that coronary atherosclerosis and coronary artery spasm are the key factors mediating MB and MI.^[24]^

### 4.4. Coronary atherosclerosis

It was shown that the vast majority of coronary atherosclerotic plaques were seen proximal to MB, which were identified by Ge et al,^[25,26]^ and the reason for its formation may be related to the fact that the expression levels of active substances such as nitric oxide synthase, endothelin-1, and angiotensin-converting enzyme are elevated proximally to MB as compared to those in the MB.^[27]^ It has also been analyzed that this may be related to the fact that MB are protected from the development of atherosclerosis by low tensile stress, high shear stress and reduced coronary artery wall tension,^[28,29]^ and that the vascular endothelial cells in the region of MB tend to have a fusiform shape, which proves that they are being under a high shear stress, whereas the polygonal distribution of the vascular endothelial cells in the other regions suggests that they have a lower shear stress.^[30]^ Increased shear stress appears to reduce monocyte adhesion and lipid transfer in the vessel wall, thereby reducing the risk of atherosclerosis.^[31]^ Nevertheless, Robbert J de Winter et al^[32]^ identified a case with coronary atherosclerotic plaques in the region of a MB by intracoronary vascular ultrasound, which opens the possibility of MI. Ramos et al^[33]^ reported a case of acute MI, in which an autopsy revealed a MB in the patient, as well as atherosclerotic plaque was found proximal to the bridge, and it was hypothesized that the cause of death was rupture of the plaque to form an occlusive thrombus, resulting in MI. However, it is still scarce on the cause of the absence of atherosclerotic plaques at the distal end of the MB.

### 4.5. Coronary artery spasm

MB can increase the risk of developing coronary artery spasm,^[34]^ which has been shown to be an important cause of MI and non-obstructive coronary arteries.^[35,36]^ Hiroki Teragawa et al^[37]^ reported a case of non-obstructive coronary MI and detected the presence of coronary artery spasm in the patient MB segments by spasm provocation test, which may be related to the constrictive kinking of the vessels leading to their endothelial dysfunction and increased local vascular reactivity to systemic vasoconstrictor stimulation.^[27]^ Purumeh Nam et al^[38]^ performed acetylcholine stimulation test and found that the incidence of coronary artery spasm was 59.1%, and patients with MB combined coronary artery spasm had a highrate of angina recurrence, proving that MB are one of the important risk factors for coronary artery spasm.

### 4.6. Diagnostic methods

Coronary angiography (CAG). The CAG typically shows systolic stenosis with complete or partial recovery of the diastolic vessels. This phenomenon was called the “milking effect.” If nitroglycerin or other vasodilating drugs were injected into the coronary vessels, the non-bridging coronary segments adjacent to the MB could be dilated, aggravating the systolic stenosis of the MB.^[8]^

Coronary CT angiography (CCTA). CCTA is one of the most generally used noninvasive examinations for the diagnosis of MB, which can clearly visualize the coronary vessel lumen, vessel wall and myocardial wall. Therefore, it has a strong accuracy for clarifying the anatomical features of MB. According to CCTA, MB can be classified into 3 categories. MB covering coronary arteries with a thickness of 1~2 mm are shallow MB. 2~5 mm are deep MB. And over 5 mm are very deep MB. One study confirmed that the detection rate of MB by CCTA was 58%,^[39]^ and its anatomical evaluation for the location, length, and depth of MB was superior to intravascular ultrasonography.^[40]^

Intravascular ultrasound (IVUS). IVUS is a technique that allows the accurate assessment of the vascular anatomy, allowing the observation of the length, depth, and compression of the vessels in the MB, as well as the combination of atherosclerotic plaques in the coronary arteries.^[41]^ The highly characteristic systolic compression and delayed diastolic relaxation of MB can be clearly visualized by IVUS, as well as the specific “meniscus,” which is located between the epicardium and the extravascular elastic membrane.^[25]^ Thus, IVUS has the unique ability to provide anatomical and functional information about MB, which has a high diagnostic value and is widely used in clinical practice.

Multidetector computed tomography (MDCT). MDCT is currently a rapid and adequate test for the diagnosis of MB, which allows the evaluation of the coronary arteries and their relationship with other anatomical structures.^[42]^ As a noninvasive test, it is comparable to IVUS in terms of accuracy and can be considered as an alternative to CAG in the diagnosis of MB.^[43]^ This test performs image reconstruction after scanning, and MB is diagnosed when coronary arteries are covered by myocardium of varying thickness, with narrowing of the lumen during systole and re-dilation of the lumen during diastole.^[44]^

Myocardial perfusion imaging (SPECT). SPECT is a modality to assess cardiac function that can preferentially detect the most severely compressed segments of MB and has a high sensitivity for moderate or greater myocardial ischemia. However, a proportion of patients with MB tend to present asymptomatic or mild myocardial ischemia affecting only a small portion of myocardium distributed within the bridge segment, when SPECT tends to show a lower sensitivity in detection.^[45]^

In addition, Elham Avard et al^[46]^ demonstrated that using radiomics analysis on non-contrast cine cardiac magnetic resonance images enables to accurately detect MI, which could potentially be used as an alternative diagnostic method for Late Gadolinium Enhancement Cardiac Magnetic Resonance (LGE-CMR). Nan Zhang et al^[47]^ proposed deep learning framework on nonenhanced cardiac cine MRI enables the confirmation (presence), detection (position), and delineation (transmurality and size) of chronic MI. However, future larger-scale multicenter studies are required for full validation.

### 4.7. Treatment

Pharmacological Treatment. Pharmacologic therapy is currently applied to the majority of MB patients. At present, β-blockers have become the first-line drugs for relieving the symptoms of MB patients, which have negative chronotropic and positive effects,^[8]^ and can reduce the heart rate and vasoconstrictive force, prolong the vasodilatory time, thus increasing coronary perfusion and normalizing the ST segment of the electrocardiogram, and further improving the symptoms of the patients and the situation of myocardial ischemia.^[48]^ However, some studies have shown that β-blockers may induce coronary artery spasm,^[49]^ so calcium channel blockers are usually applied to patients with poor tolerance of β-blockers. Some studies have shown that they may have vasodilatory effects, which is beneficial to patients with vasospasm, such as verapamil and diltiazem.^[3]^ It has been suggested that clinics may perform acetylcholine provocation tests in patients with MB, prompting the introduction of calcium channel blockers in patients with evidence of vasospasm and a poorer prognosis, which is more helpful in guiding the management of patients with MB.^[50]^ Ivabradine is the new and only sinus node hyperpolarization-activated cyclic nucleotide-gated inhibitor that reduces heart rate in patients, and is mainly used in patients who cannot tolerate β-blockers or calcium channel blockers, or who fail to achieve a normal heart rate despite treatment, and is currently used only as a second-line agent to alleviate the symptoms of MB.^[51,52]^

Percutaneous coronary intervention. Percutaneous coronary intervention is a therapeutic approach for symptomatic patients with MB who are also intolerant to medications or have already developed MI. Haager et al^[53]^ performed intracoronary stenting within MB in 11 patients with myocardial ischemia and no other cardiac disease. After 7 weeks, 5 patients developed varying degrees of in-stent stenosis, and 4 of them underwent target revascularization, all patients showed good angiographic results after 6 months, and all patients were free of angina pectoris and adverse events 2 years thereafter. However, compression of the stent by the MB creates a potential risk of stent fracture,^[54]^ and the in-stent restenosis rate ranges from 36% to 75% for bare-metal stents and 18% to 25% for drug-eluting stents,^[55]^ therefore, it is not currently the treatment of choice.

Surgical treatment. Surgical treatment is recommended for patients with more severe symptoms who also have poor results with oral medication. There are 2 main surgical approaches, supra-arterial myotomy and CABG. Huang et al^[56]^ reported 11 patients who underwent supra-arterial myotomy and CABG. Only 2 had atypical chest pain and the others were asymptomatic at the 3-year follow-up. None had major adverse cardiac events. Wu et al^[57]^ selected 31 patients (24 males and 7 females) with MB for surgical treatment, 15 of whom underwent supra-arterial myotomy and 16 underwent CABG, with preoperative cardiac function graded as NYHA class I in 5 cases, NYHA class II in 18 cases, and NYHA class III in 8 cases. Angiographic studies in 21 patients (68%) after 3 to 115 months of follow-up showed coronary artery blood flow and myocardial perfusion were restored and all patients were asymptomatic and currently in NYHA class I-II. However, in the presence of MB, systolic coronary flow is reduced by only 15% and in diastole flow is almost normal. So there is a risk of competing blood flow, impaired bridge vessel function, or even bridge vessel occlusion^[58]^

### 4.8. Limitation

This study analyzed the hotspots and future trends of MB by bibliometric methods, but it undeniably still has some limitations. First, the currently used bibliometric software has certain limitations, which makes it difficult to combine multiple databases such as PubMed, Embase, Scopus, etc for analysis. So only WoSCC database was selected for this study. However, WoSCC database is currently recognized as one of the most authoritative data platforms for scientific literature retrieval. We will try to use more databases for analysis in the future. Second, we only selected English literature, which may lead to some relevant literature not being included in this study. If all the literature had been included in this study, the results would have been more convincing. In the end, we may reduce some of the accuracy when analyzing the data, such as when analyzing the co-cited authors, VOSviewer showed the abbreviations of the authors’ names. There may be cases of renaming, thus causing a lack of accuracy. So we will further optimize the bibliographic approach in the future.

## 5. Conclusion

In conclusion, it is not difficult to see that more and more researchers have begun to pay attention to the study of MB combined with MI all over the world, which has an important research value, and the bibliometric analysis can help to analyze the research process and frontiers in the last 30 years, so as to find out the direction of future research. The current hotspots of MB combined with MI research include the pathology and pathophysiology, diagnostic methods, and treatments, etc. Therefore, we hope that each country can fully cooperate with each other and make contributions to the health of the whole mankind.

## Acknowledgments

The authors would like to express their appreciation to Professor CM Chen, who invented CiteSpace, which is free to use.

## Author contributions

**Conceptualization:** Yuejuan Cao.

**Data curation:** Haiyuan Zhang, Yuejuan Cao.

**Formal analysis:** Haiyuan Zhang.

**Supervision:** Yuejuan Cao.

**Writing – original draft:** Haiyuan Zhang.

**Writing – review & editing:** Yuejuan Cao.
